# Micro/meso scale investigations on F-slag sand developed with synergistic use of fly ash and slag

**DOI:** 10.1038/s41598-026-43476-x

**Published:** 2026-03-10

**Authors:** Kummari Sekhar, Bendadi Hanumantha Rao, Vesna Zalar Serjun

**Affiliations:** 1 Research Scholar, School of Infrastructure, Indian Institute of Technology, Bhubaneswar, Odisha, 752 050 India; 2 School of Infrastructure, Indian Institute of Technology Bhubaneswar, Odisha, 752050 India; 3 Department of Materials, Slovenian National Building and Civil Engineering Institute, 1000 Ljubljana, Slovenia

**Keywords:** F-Slag sand, geopolymer-assisted pelletization, artificial fine aggregates, fly ash-slag synergy, micro-meso scale characterization, disc pelletizer, Engineering, Environmental sciences, Materials science

## Abstract

The present study propounds development of an artificial sand, with a prime aim of exploring a potential substitute for natural river sand. The artificial sand, named F-Slag sand, within the particle size range of 4.75 − 0.075 mm, is developed with the combined use of fly ash (FA) and ground granulated blast furnace slag (GGBS) as binder solids (BS) blended in proportions of 90:10, 80:20, 70:30, 60:40, and 50:50. Pelletization, by employing a custom designed disc pelletizer equipment, and geopolymerization, a process of binding and bonding of FA and GGBS particles to form sand size fractions, techniques are adopted to synthesize F-slag sand. Pelletizer speed of 15 rpm, pelletization duration of 10 min, and alkali activator solution to BS ratio of 0.06 are found optimal to produce the sand. A comprehensive micro/meso-analysis, including 3D computed tomography scan, scanning electron microscopy, X-ray diffraction, Brunauer-Emmett-Teller analysis, and Fourier transform infrared spectroscopy are performed on F-slag sand and attempts are made to interpret macro-applications of it. Demonstrably, the synergistic use of GGBS and FA has completely alleviated the need for elevated curing of sand. The gradational analysis revealed that more than 90% of BS are converted into sand size fractions (4.75 − 0.075 mm). F-slag sand exhibited specific gravity of 2.1, water absorption of 17%, permeability 4.2 × 10^− 3^ cm/sec, angle of internal friction of 38°, and crushing value of 14.8%. In comparison, river sand showed 2.6, 4%, 3.6 × 10^− 3^ cm/sec, 39° and 6.3% respectively. The loose and compacted bulk densities of F-slag sand are measured at 698 and 991 kg/m^3^, which are significantly lower vis-à-vis of river sand (1546 and 1728 kg/m^3^). Micro-analysis confirms dense, well-bonded particles with meso–macroporous connectivity, indicating enhanced mechanical stability and durability. Moreover, leaching and environmental risk analysis affirms that F-Slag sand poses no ecological threat. The outcome of the study, proposition of a novel construction material, elucidates that F-slag sand could be a potential substitute for river sand in building (concrete, mortar, plastering, lightweight structures), mining (mine backfilling) and environmental (drainage & filter) applications.

## Introduction

Sustainability of construction industry is largely pivoted on advancements in novel materials that alleviate the environmental impact. As the global construction sector experiences unprecedented expansion, demand for fine aggregates, mainly natural river sand, has skyrocketed. This escalated demand has resulted in over exploitation and thus, rapid depletion of natural river sand reserves in both developing and developed nations, triggering severe environmental consequences^1^. Notably, global sand consumption will reach around 82 billion tons by 2060^2^. In India alone, the annual consumption of river sand is estimated at 140 million tons, aggravating ecological repercussions^3^. On the other hand, the massive generation of industrial by-products poses a grave threat to the environment. Despite their vast abundance, utilization remains limited, intensifying ecological issues^4,5^. Address of this dual challenge requires innovative technologies promoting large-scale repurposing of industrial by-products into value-added materials.

Among many industrial by-products, fly ash (FA) and ground granulated blast furnace slag (GGBS) stand out as promising materials for the sustainable construction purposes. The superior properties of FA, such as low heat of hydration, chemical resistance, reduced CO_2_ emissions, and improved workability, make it an ideal component for concrete and construction applications^6^. On similar lines, GGBS, rich in calcium, significantly enhances the mechanical strength, durability, and sustainability of concrete structures, earning LEED (Leadership in Energy and Environmental Design) certification^7,8^.

A promising avenue that addresses the dual challenge of river sand scarcity with concurrent industrial by-product utilization is the production of artificial fine aggregates i.e. artificial sand, as a sustainable alternative for river sand. In this direction, Sudam et al.^9^ and Rao and Acharya^10^ have developed geopolymer sand from FA by hand crushing. Although this method produces sand-sized particles, it is energy-intensive, requires significant manual intervention, and results in irregular shape of particles, thereby limiting its practicality for large-scale applications. Gnanadurai et al.^11^ have developed synthetic sand using a combination of FA and GGBS by hand mixing. However, this approach is limited to laboratory-scale production and is not feasible for scale-up to manufacturing-level applications. Earlier studies successfully demonstrated the pelletization technique for producing coarse aggregates with particle sizes greater than 10 mm^12,13^. Recently, Sekhar and Rao^14^ have made a significant advancement in manufacturing artificial sand solely from fly ash by resorting to geopolymerization and cold-bonded pelletization techniques. The proposed technology is fully automatized and it completely alleviates the manual intervention, including hand crushing and mixing. Further, the earlier proposed methods require elevated temperature for curing sand and that the median size of precursor materials only can be used for making the sand^15^. However, the outcome of these research highlight that blending of GGBS with FA seems to have notable benefits, including enhanced compressive strength and elimination of energy-intensive oven curing^11^.

The novelty of the present study lies in synthesis and development of artificial sand with the synergistic utilization of FA and GGBS as binder solids (BS) employing a custom-designed disc pelletizer equipment. The BS are activated with alkali solutions, such as sodium silicate and sodium hydroxide, in line with the philosophy of geopolymer technology. A bespoke disc pelletizer is employed to transform BS into fine aggregates of a desirable size range of river sand (i.e. 4.75 to 0.075 mm). It is appreciated that the whole process of F-slag sand synthesis completely circumvents the need for elevated curing, such step is compulsory when used fly ash alone as a precursor material. The ambient curing of artificial sand is meritorious, as it can aid to scale-up the manufacturing process. The developed sand particles are comprehensively characterized for their physical, mechanical, leaching, and thermal behaviour. Micro-scale investigations include, scanning electron microscopy, X-ray diffraction, and Fourier transform infrared spectroscopy, along with meso-scale analyses such as Brunauer-Emmett-Teller surface area measurement and nitrogen adsorption, are performed on F-slag sand. Besides, 3D computed tomography studies are performed to endorse the macro-performance of sand. The proposed novel technique of developing artificial sand tackles the dual issues of the burgeoning need for natural river sand and growing demand for fine aggregates. The synthesized F-slag sand offers practical applications in concrete, mortar, plastering, lightweight structures, drainage & filtration, and backfilling, linking sustainable waste utilization with construction needs.

## Materials and testing methodology

### Materials

Fly ash, FA, was obtained from National Thermal Power Corporation Limited, Kaniha, while GGBS was procured from Bhubaneswar, Odisha, India. The selection of FA is motivated by their rich aluminosilicate and pozzolanic phases, which are essential precursors required for the geopolymerization reaction to occur^16,17^. The incorporation of GGBS enhances the binding and strength development by its affluent calcium content^18^. The physical properties of FA and GGBS were determined in compliance with the relevant standards. Particle size analysis was done in accordance with ASTM D6913/D6913M-17 guidelines^19^. Specific gravity and bulk density were determined following ASTM D854-14 and C29/29 M-17a standards^20,21^.

Extensive literature suggests that the combined application of sodium silicate (Na_2_SiO_3_) and sodium hydroxide (NaOH) solutions significantly enhances the compressive strength of geopolymer aggregates. Moreover, this approach is more cost-effective than NaOH alone as the activator^22^. In light of these findings, the present study utilized alkaline activator solutions (AAS) such as Na_2_SiO_3_ and 97% pure NaOH flakes, procured from Bhubaneswar, Odisha, India. The AAS consist of a mixture of Na_2_SiO_3_ and NaOH solutions. The ratio of Na_2_SiO_3_ to NaOH solution is fixed at 1.5, as per the literature recommendation^23,24^. Excessive NaOH concentrations could adversely affect aggregate properties, so the NaOH concentration was fixed at 6 M, striking a balance between production cost and performance^25^. To prepare 6 M NaOH solution, 247 g of NaOH flakes were dissolved in one liter distilled water. The solution was then thoroughly stirred to ensure complete dissolution and subsequently left undisturbed for 24 h, allowing the exothermic heat to dissipate before its use.

### Development of F-Slag sand

The method of developing F-Slag sand adopted in this study follows the process and custom designed disc pelletizer proposed by Sekhar and Rao^14^. This innovative approach leverages two industrial by-products, FA and GGBS, mixed by mass in proportions of 90:10, 80:20, 70:30, 60:40, and 50:50 to develop a single product i.e. F-Slag sand. The making process begins by feeding air-dried FA and GGBS of 1 kg into the disc pelletizer and dry mixing them for 5 min. Subsequently, AAS (i.e. a mixture of NaOH and Na_2_SiO_3_) were slowly sprayed onto BS, and the rotation was continued for 3–5 min to ensure the formation of thin film around BS particles. Based on the extensive trials, optimal operating conditions such as pelletizer tilt angle and speed were set at 50 degrees and 15 rpm. AAS/BS ratio was fixed at 0.06, based on the trials. Table 1 summarizes the experimental trials along with the designations used for developing F-Slag sand. As the moistened particles hit and roll within the drum, bonding forces at contact points gradually strengthen, forming sand-sized granules. After a pelletization duration of 10 min, sand was collected and cured under ambient conditions (27 ± 1 °C) for 28 days.

**Table 1:** Details of the trials with designations to develop F-Slag sand.

Trial	Designation	FA: GGBS ratio	AAS/BS ratio	Speed of pelletizer (rpm)	Time of pelletization (min)
T1	90FA10GGBS0.06R15P_s_10t_p_	90:10	0.06	15	10
T2	80FA20GGBS0.06R15P_s_10t_p_	80:20			
T3	70FA30GGBS0.06R15P_s_10t_p_	70:30			
T4	60FA40GGBS0.06R15P_s_10t_p_	60:40			
T5	50FA50GGBS0.06R15P_s_10t_p_	50:50			

### Characterization of F-Slag sand

Phyisco-mechanical properties of F-Slag sand (T4) were evaluated on the whole sand (4.75 mm to 0.075 mm) including gradation, specific gravity, water absorption, density, permeability, shear strength, and pH, as per the relevant ASTM standards C136/C136 M–19, C128, C29, D2434, and D3080^21,26–30^. Additionally, crushing strength of F-Slag and river sand was measured, as per IS 2386 Part 4 standard^31^. The obtained properties of F-Slag sand were compared vis-à-vis with river sand to gain deeper insights into its applicability.

#### Microstructure and morphological properties

Morphological analysis was performed using a MERLIN compacted field emission scanning electron microscope (FE-SEM) (make, ZEISS FEI Quanta 25, Berlin) to delineate the aggregation of F-Slag sand particles. Before conducting the analysis, particles were sputtered with gold using a Q150R ES sputter coater in order to capture best particle morphology.

The Brunauer, Emmet, and Teller (BET) surface area, pore size, and pore size distribution in the range of 2–300 nm were determined in accordance with ISO 15901-2 (2022)^32^, using ASAP 2020 equipment (Micromeritics, Norcross, Georgia). Nitrogen gas sorption was employed, and the samples were degassed under vacuum at 105 °C with a rate of 0.67 kPa/s to a final pressure of 26.7 Pa. Degassing was carried out on preheated samples at 105 °C with an equilibrium exposure interval of 10 s (p/p0 = 1.000000). The BET surface area was calculated in the relative pressure range of 0.05–0.3. The pore size distribution was evaluated using the Barrett–Joyner–Halenda (BJH) method with the Halsey equation, which relates pore size to the critical condensation pressure. Pore analysis was conducted on the adsorption branch of the isotherm.

In addition to conventional morphological characterization, advanced 3D micro-computed tomography (CT) analysis was employed to understand the internal structure of F-Slag sand particles with high precision. The scanning system utilized a Hamamatsu L10711-19 X-ray tube coupled with Varian flat panel detector. This enabled non-destructive visualization of pore distribution, particle morphology, grain distribution, and spatial connectivity within the material, unveiling microstructural features crucial to understanding the material’s functional behavior and potential performance in construction applications. To further enhance the analysis, 3D rendering techniques were applied to map the spatial orientation and morphology of pores, while Dragonfly software was utilized for porosity assessment, delivering a robust approach for evaluating the material integrity.

#### Analysis of phase composition

Phase composition was established by powder X-ray diffraction (XRD) analysis, using a D8 Advance X-ray powder diffractometer fitted with a high-resolution LYNXEYE detector and employing copper (Cu-Kα) radiation. Scanning was performed at 40 mA current and 30 kV voltage, covering a 2θ range from 5° to 85°, with a step size of 0.2. The X’Pert High Score Plus software was utilized to characterize the mineral phases.

In order to perform a detailed mineralogical composition of F-Slag sand, some other complementary test methods were used, such as Fourier transform infrared (FTIR) and thermogravimetric (TG) analysis with quadrupole mass spectroscopy (QMS). FTIR spectroscopy was performed with internal reflection configuration using the attenuated total reflectance (ATR) technique. FTIR analyses were carried out with a PerkinElmer Spectrum 100 FTIR spectrometer (Shelton, CT, USA). Spectra were recorded with a spectral resolution of 4 cm⁻¹ in the range 4000–450 cm⁻¹. Each sample was scanned 64 times. TG analyses were performed in the range from room temperature to 800 °C at a heating rate of 5 K/min, under an air flow of 50 mL/min. Alumina crucibles (150 µL) were used, with an initial sample mass of 10 mg, and the measurements were carried out in duplicate. Prior to heating, the samples were purged with air (50 mL/min) for 20 min. This “isothermal” step was not considered, i.e. the TG curve was recorded from the onset of heating, with time starting at 0 min. The measurements were conducted on a Mettler Toledo TGA/DSC 1 system coupled with a Thermostar QMS.

#### Leaching analysis

To evaluate the environmental acceptability of the developed F-Slag sand, Toxicity Characteristic Leaching Procedure (TCLP) was carried out in accordance with USEPA Method 1311^33^. Key potentially toxic elements (PTEs) such as Arsenic (As), Copper (Cu), Chromium (Cr), Cadmium(Cd), Zinc (Zn), Lead (Pb), Nickel (Ni), and Mercury (Hg) were quantified using an ICP flame photometer with optical spectroscopy. Additionally, concentrations of Calcium (Ca), Potassium (K), Magnesium (Mg), Iron (Fe), and Sodium (Na) were determined. The leached concentrations were compared against various international regulatory thresholds such as MoEF&CC, Government of India^34^, VROM^35^, CONAMA^36^, ABNT NBR^37^, and USEPA^38^, to ensure environmental compliance and safe usage.

#### Environmental risk assessment

Two widely recognized assessment methods, such as the single factor index (P_i_) and the potential ecological risk index (E_i_), were employed to assess the potential environmental impact of heavy metals in F-Slag sand on water and soil systems. The study focused on eight key elements: As, Cu, Cr, Cd, Zn, Pb, Ni, and Hg. To ensure a comprehensive evaluation, toxicity response coefficients and standard reference values for these elements were incorporated, following the established benchmarks outlined in the literature^39,40^.

## Results and discussion

### Characterization of precursor materials

**Fig. 1:**
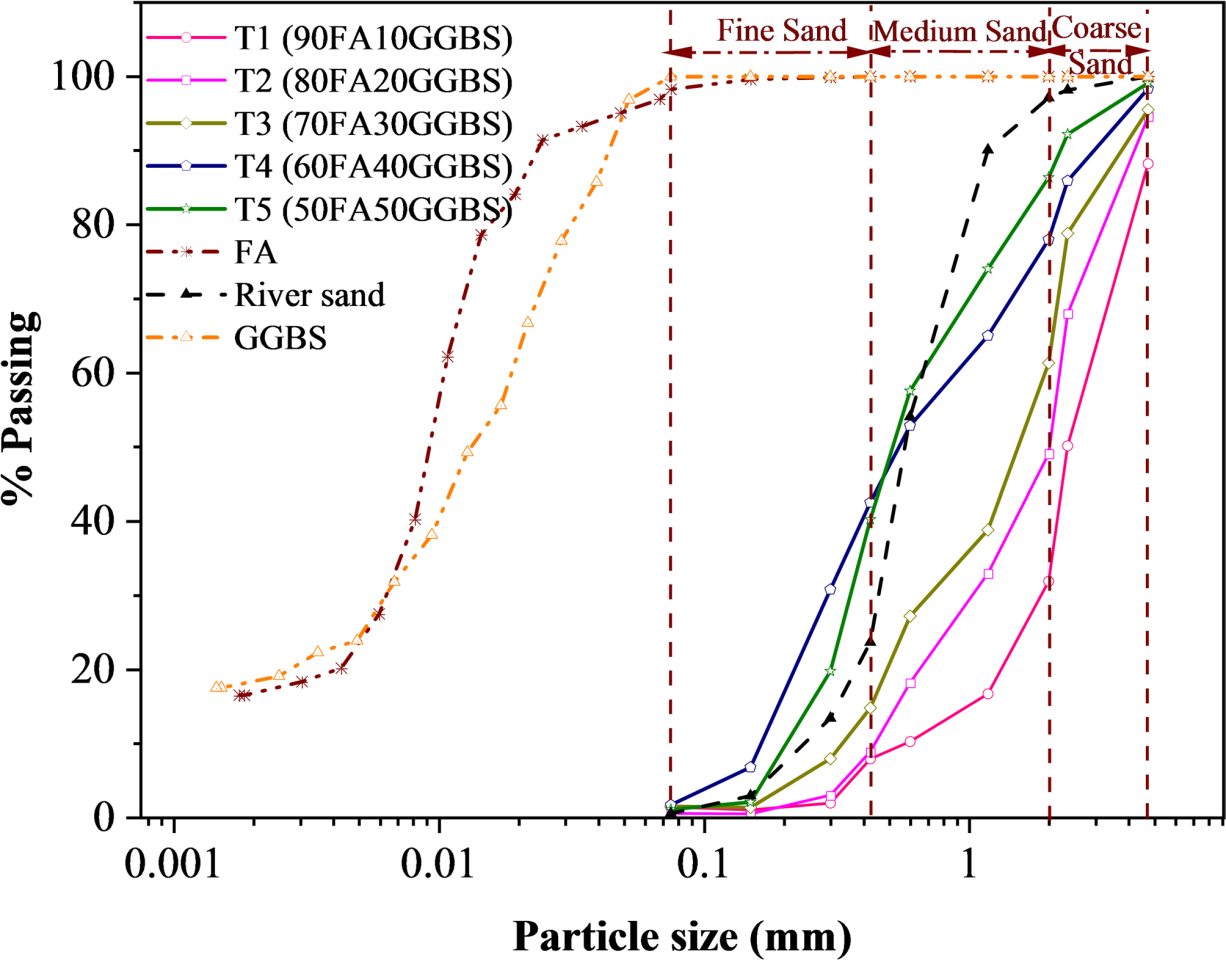
Gradational curves of F-Slag sand (T1-T5) and raw materials (FA, GGBS, and river sand).

Figure 1 depicts the particle size distribution of precursor materials i.e. FA and GGBS. The graph shows that both these materials are dominantly consist silt-sized particles. FA contains 2% sand-sized, 81% silt-sized, and 17% clay-sized fractions, while GGBS comprises of 82% silt-sized and 18% clay-sized fractions. The specific gravity of these materials is measured as 2.2 and 2.7 respectively. The loose and compacted densities (kg/m^3^) are measured as 820 and 1243 for FA, and 875 and 1326 for slag. In terms of visual characterization, FA is found in greyish-white color while GGBS in white color.

### Particle size distribution of F-Slag sand

Figure 1 depicts the gradational curves (5 trials, T1-T5) of F-Slag sand synthesized for variable propositions of binder solids (FA & GGBS). Gradation curves of river sand along with precursor materials are superimposed in the graph for illustrative purpose. The designation of each trial, say 90FA10GGBS, indicates 90% FA blended with 10% GGBS, as detailed in Table 1. Figure 1 highlights a distinct gradation gap between the precursor materials and their derived product, showcasing the successful synthesis of F-Slag sand. The percent conversion of precursor materials into F-Slag sand can also be observed from Fig. 1. For a better understanding, each trial is fractionized into gravel (> 4.75 mm) and sand (4.75 –0.075 mm) components. The sand fraction is further divided into coarse (4.75–2 mm), medium (2–0.425 mm), and fine sizes (0.425 –0.075 mm).

As evident from Fig. 1, fine sand fraction varies from 7 to 41%, medium sand from 24 to 46%, and coarse sand from 12 to 56%. Notably, as GGBS content increases from 10 to 50% (T1 to T5), the overall sand fraction rose from 87.6 to 98.8%, while the gravel and coarse sand fractions declined from 11.7 to 0.9% and from 56.3 to 12.7% respectively. Incidentally, variations are observed only in trends of fine and medium sand fractions. From T1 to T3, the fine and medium sand fractions increased steadily from 7.2 to 14.2% and from 24 to 46.5%. However, for T4, the proportions shifted, with fine sand reaching 41.8% and medium sand reduced to 35.5%, showcasing a nuanced trend influenced by mix proportions. Moreover, the fineness modulus of the developed F-Slag sand is determined as 4.3, 3.8, 3.5, 2.6, and 2.5 for the combinations T1 to T5 respectively. ASTM C33^41^ specifies a fineness modulus between 2.3 and 3.1 for fine aggregates intended for concrete applications. Among the tested combinations, T4 and T5 fall within this range, underscoring their suitability for concrete production. The results aforementioned demonstrate that artificial sand can be manufactured with the synergistic use of two distinct industrial by-products, such as FA and GGBS. The findings highlight the importance of optimizing the BS ratio to achieve the desirable sand gradations tailored to a specific requirement.

Moreover, the incorporation of GGBS contributed positively for sand formation by enhancing particle bonding and mechanical stability during pelletization. The angular and rough surface texture of GGBS particles improved interparticle friction and mechanical interlocking, facilitating the formation of stable sand-sized granules. In addition, the presence of reactive calcium- and silica-rich phases in GGBS promoted secondary gel formation under alkaline activation, which strengthened the bonding between FA and GGBS particles and reduced aggregate breakage during rolling. The relatively higher density of slag also aided in controlling excessive fines generation resulting in sand fraction. Overall, slag acted as both a physical stabilizer and a supplementary reactive component, thereby improving sand formation efficiency and particle integrity.

### Properties of F-Slag sand

The specific gravity of F-Slag sand is measured as 2.1, which is close to river sand (2.6) and aligns with previous studies on geopolymer-based artificial sand^14^. The water absorption of F-Slag sand is measured as 17%, which is notably higher than that of river sand (4%) but corroborates with the literature data^9,11^. The permeability (cm/s) of river and F-slag sand is measured as 3.6 × 10^− 3^ and 4.2 × 10^− 3^, respectively, which are nearly the same, indicating similar drainage behavior and particle size. Bulk density measurements reveal that F-Slag sand has loose and compacted densities (kg/m³) of 698 and 991, which are considerably lower than river sand (1546 and 1728 kg/m^3^), beneficial in reducing the dead loads. Shear strength property such as angle of internal friction is measured for F-slag sand as 38°, which is quite close to river sand of 39°, aligning well with literature findings^14^. The pH of F-Slag and river sand is measured as 12.1 and 7.7 respectively. F-Slag sand exhibited crushing strength value of 14.8% while river sand showed 6.3%. Both these values are excellently within the permissible limit of 30% for the construction grade sand^31^. The relatively low crushing value of F-Slag sand outlines its impressive strength, attributed to the synergistic effect of FA and GGBS^11,15^. Interpretation of these results underline the prospective of F-Slag sand as a potential substitute for river sand in fine aggregate category of construction materials.

### Morphological and mineralogical studies

**Fig. 2:**
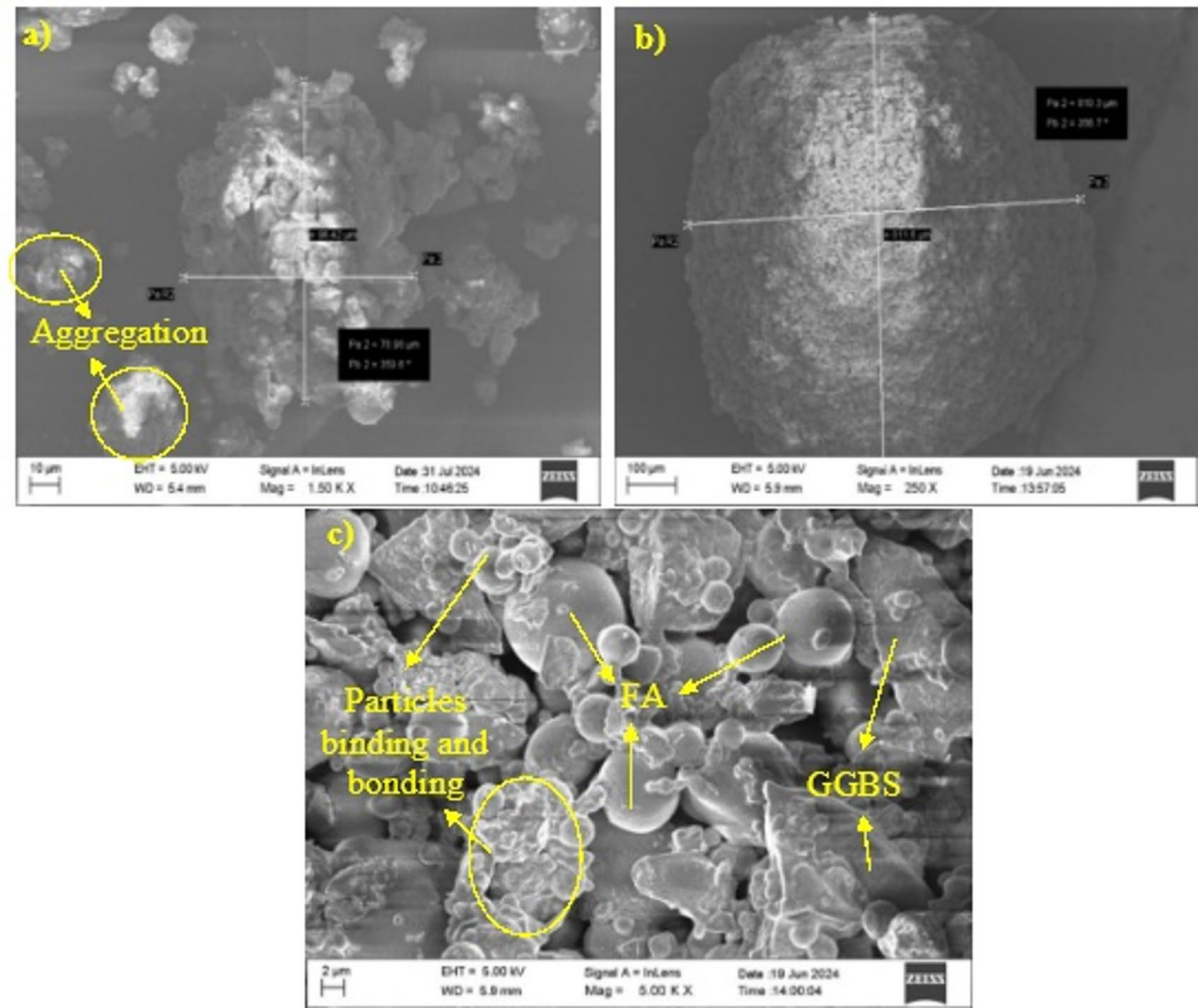
SEM images of **a-c**) F-Slag sand particles.

Figure 2 depicts the morphology of F-slag sand. A close observation of images (Fig. 2(a-c)) discloses bonding of angular (GGBS) and spherical (FA) grains together forming a single sand particle with a diameter of more than 78 μm. Evidently, adherence of FA particles to GGBS can be seen from these images. This morphology might have emerged as a result of conglomeration of FA and GGBS grains via bonding and binding that would have occurred due to the geopolymerization. Whereas, the formation of sand particles could be attributed to the pelletization process coupled with alkali activation, triggering dense packing of Si-O-Al-O units by reaction products due to the dissolution, reorientation, and polycondensation^39^.

**Fig. 3:**
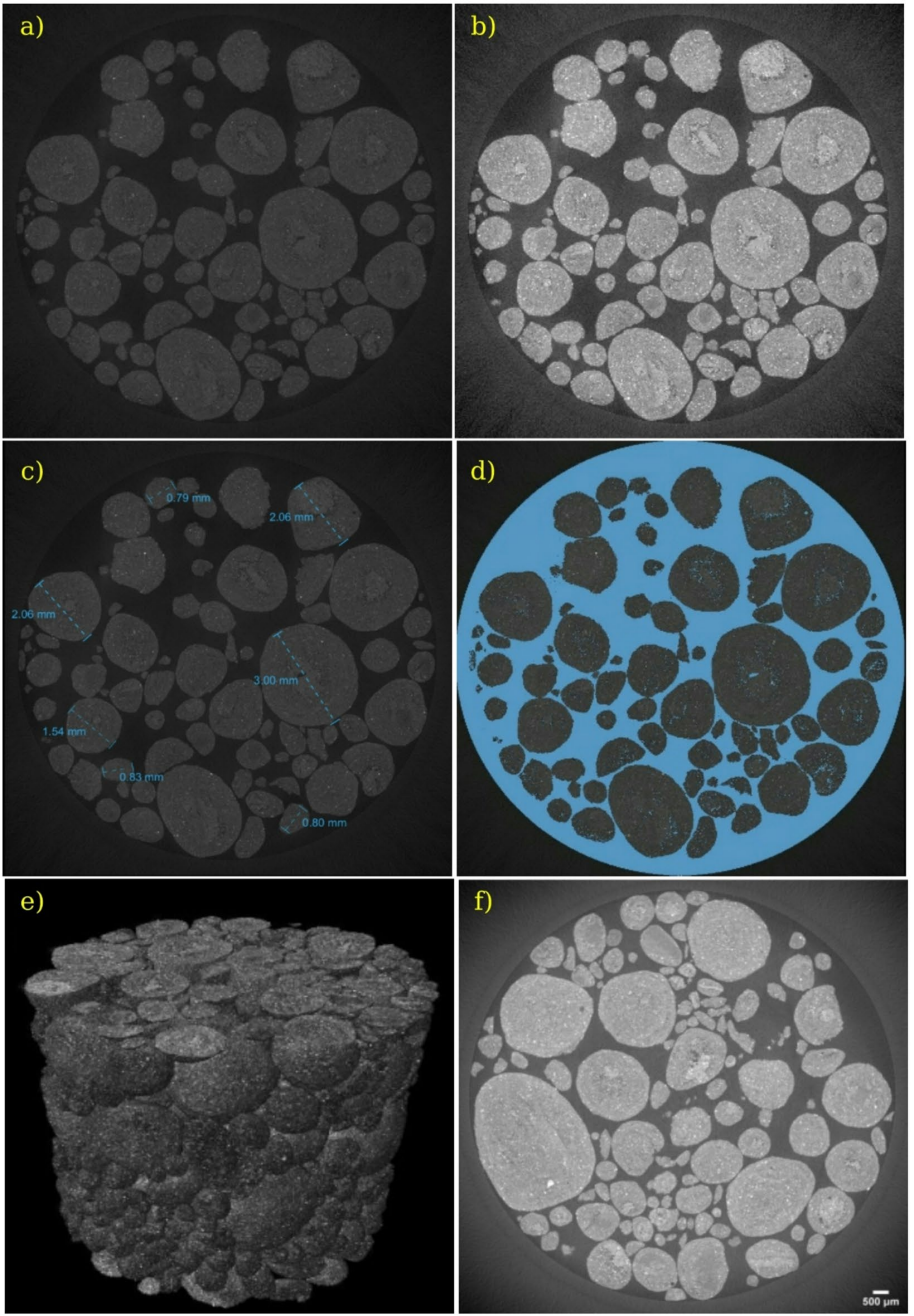
CT scan images of F-Slag sand **a**) original image, **b**) brightness adjusted, **c**) various particle sizes, **d**) Otsu segmented image, **e**) 3D rendering top view, and **f**) 3D rendering bottom view of the axial plane.

3D micro CT scan analysis, depicted in Fig. 3, offers a comprehensive visualization of the internal structure and particle distribution of F-Slag particles. Figure 3a displays a single original CT slice, capturing the raw microstructural features while Fig. 3b shows the brightness-adjusted image, enhancing contrast and allowing clearer identification of particle boundaries. This enhanced image clarity aids in distinguishing finer textural details. Figure 3c illustrates the particle size distribution within the slice, with sizes ranging from 0.79 mm to 3 mm, revealing the variable gradation in sand particles. Particle sizes are determined from reconstructed CT slice images using Fiji (ImageJ) software. The maximum particle dimension is measured by drawing straight-line profiles across individual particles. The images are calibrated considering an isotropic voxel size of 0.25 mm in all directions, as determined experimentally. The measured particle sizes correspond to these calibrated line measurements. Figure 3d shows the Otsu-thresholded segmentation of the image, which isolates the solid phase from the voids and background. A detailed comparison between Fig. 3b and d highlights the presence of internal voids within the agglomerated particles. This kind of features are crucial for understanding the porosity and potential weakness zones in the developed material.

**Fig. 4:**
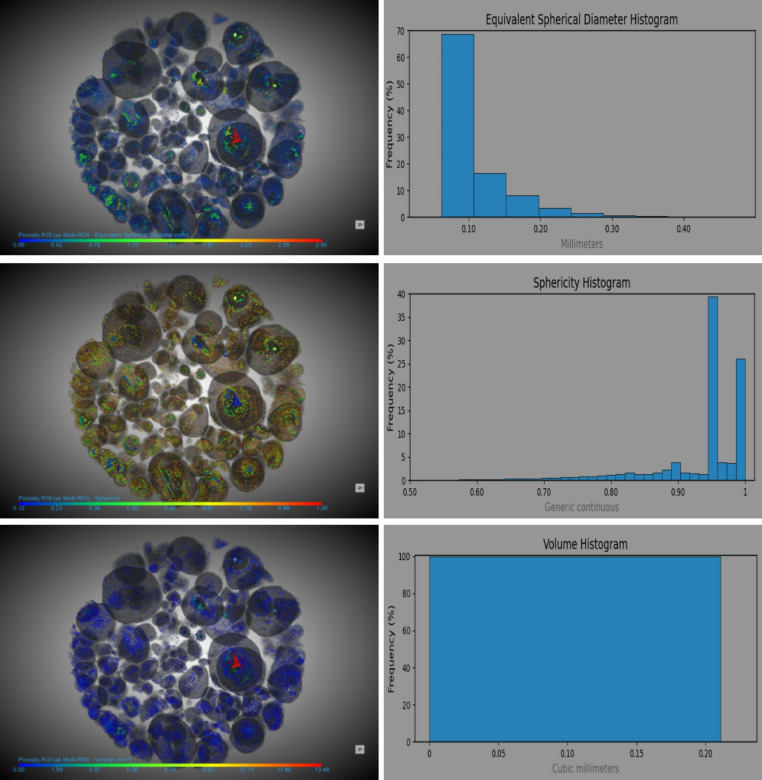
Porosity of developed F-Slag sand particles.

Figure 4 depicts the porosity analysis of developed F-Slag sand particles, integrating 3D CT scan imagery with corresponding histograms of key morphological parameters, including equivalent spherical diameter, sphericity, and pore volume. This combined visual and quantitative approach reveals a comprehensive view of F-Slag sand particles internal structure. The equivalent spherical diameter of pores predominantly ranges from 0.07 to 0.3 mm, with a peak frequency at 0.1 mm, representing 68% of the distribution. In contrast, pores around 0.2 mm have occurred infrequently, contributing to less than 5% of the total. Sphericity values range from 0.7 to nearly 1.0, with the majority of pores (39%) exhibiting a high sphericity of approximately 0.95, reflecting a microstructure dominated by nearly spherical voids. Pore volume is largely constrained within 0–0.2 mm³ range, suggesting a tight distribution of internal void spaces. These porosity characteristics are not only indicative of the microstructural precision achieved during synthesis but also correlate strongly with the physical behaviour of the material. The prevalence of small, spherical pores may explain the increased water absorption and lower bulk density observed in Sect.  3.3. However, the overall low porosity levels point to effective bonding between precursor particles in the presence of AAS, resulting in stable, sand-sized granules. When correlated with SEM images, which provide surface-level morphological validation, the CT-based porosity analysis offers a holistic understanding of the F-Slag sand’s microstructure. The uniformity in pore size and shape enhances packing density and reduces stress concentrations, promoting better mechanical interlocking when used in concrete or other construction materials.

**Fig. 5:**
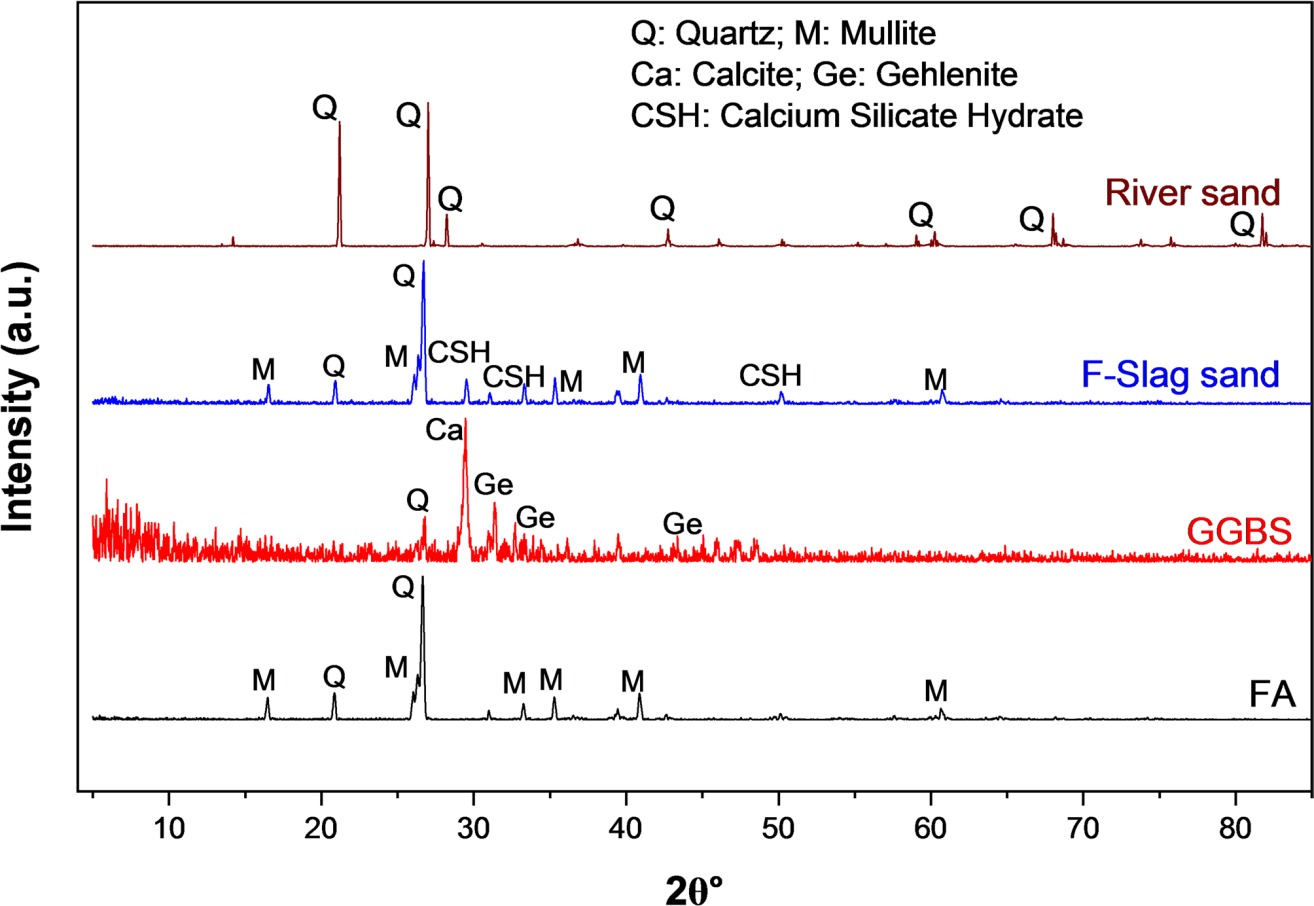
X-ray diffractograms of FA, GGBS, F-Slag, and river sand.

Figure 5 illustrates the mineralogical compositions of FA, GGBS, F-Slag, and river sand. Quartz is identified as predominant mineral in natural river sand. In FA, the key minerals identified are quartz and mullite. In F-slag sand, diffraction peaks associated with quartz and mullite are retained while the presence of amorphous background reflects the formation of geopolymer gel during alkali activation. Peaks of quartz are found at 2θ of 20.84 and 26.77 degrees. Whereas, mullite peaks occurred at 2θ of 16.53, 26.35, 35.31, 41.04, and 60.82 degrees. This amorphous geopolymer gel acts as a binding matrix that bridges FA and GGBS particles, resulting in enhanced interparticle bonding and improved structural integrity. Its non-crystalline nature promotes dense particle contact network while maintaining interconnected meso- to macroporosity, thereby enhancing mechanical stability, resistance to particle breakage during pelletization. Interestingly, the diffractogram of F-Slag sand reveals the emergence of new peaks associated with calcium silicate hydrate (CSH) gels at 2θ of 29.52, 33.26, and 50.21 degrees. These CSH formations can be attributed to the synergistic reaction between FA and GGBS in the presence of AAS, composed of NaOH and Na₂SiO_3_, wherein the calcium-rich GGBS participates in secondary hydration reactions alongside geopolymer gel formation. This confirms effective geopolymerization and enhanced binding within the F-slag sand matrix. This combined action enhances strength development and enables ambient curing of sand particles, eliminating the need of elevated curing requirement in case of fly ash based sand^15^. The formation of CSH gels in synthetic sand made of FA alone and the combination of FA and GGBS is also reported in the literature^11,42^. These CSH gels function as micro-aggregates within the geopolymer matrix, refining its microstructure^43^.

**Fig. 6:**
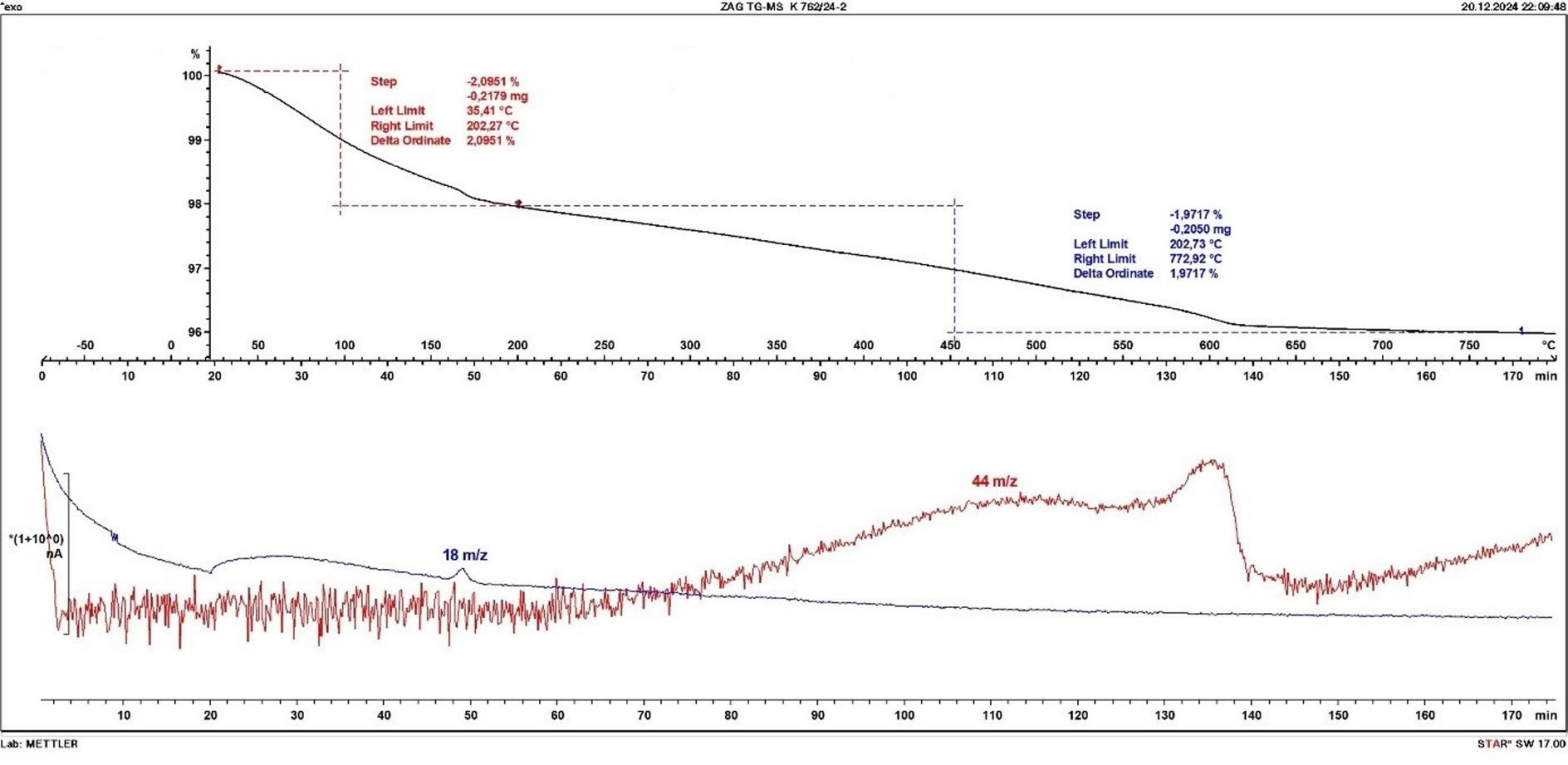
Thermogravimetric analysis curve of F-Slag sand.

The TGA curve of F-slag sand (Fig. 6) reveals its multistage weight-loss, indicating its compositional transitions with increasing temperature, reflecting characteristic of FA-GGBS blend geopolymer system^44,45^. An initial reduction of about 2.095% below 202 °C corresponds to the evaporation of physically adsorbed moisture and loosely bound surface water present within the pore structure of geopolymer matrix, as widely reported for geopolymer gels^46^. This is followed by a gradual reduction of nearly 1.971% between 202 and 772 °C, which can be ascribed to the dehydroxylation of hydrated phases and the partial breakdown of volatile constituents, corroborated by weak gas-evolution features in the coupled QMS at m/z = 18 (H_2_O) and m/z = 44 (CO_2_)^47,48^. In total, F-Slag sand records a 4.07% mass loss up to 800 °C, retaining 95.93% of its initial mass. This indicates that the material possesses substantial thermal stability, making it promising for use as an alternative fine aggregate in construction applications.

**Fig. 7:**
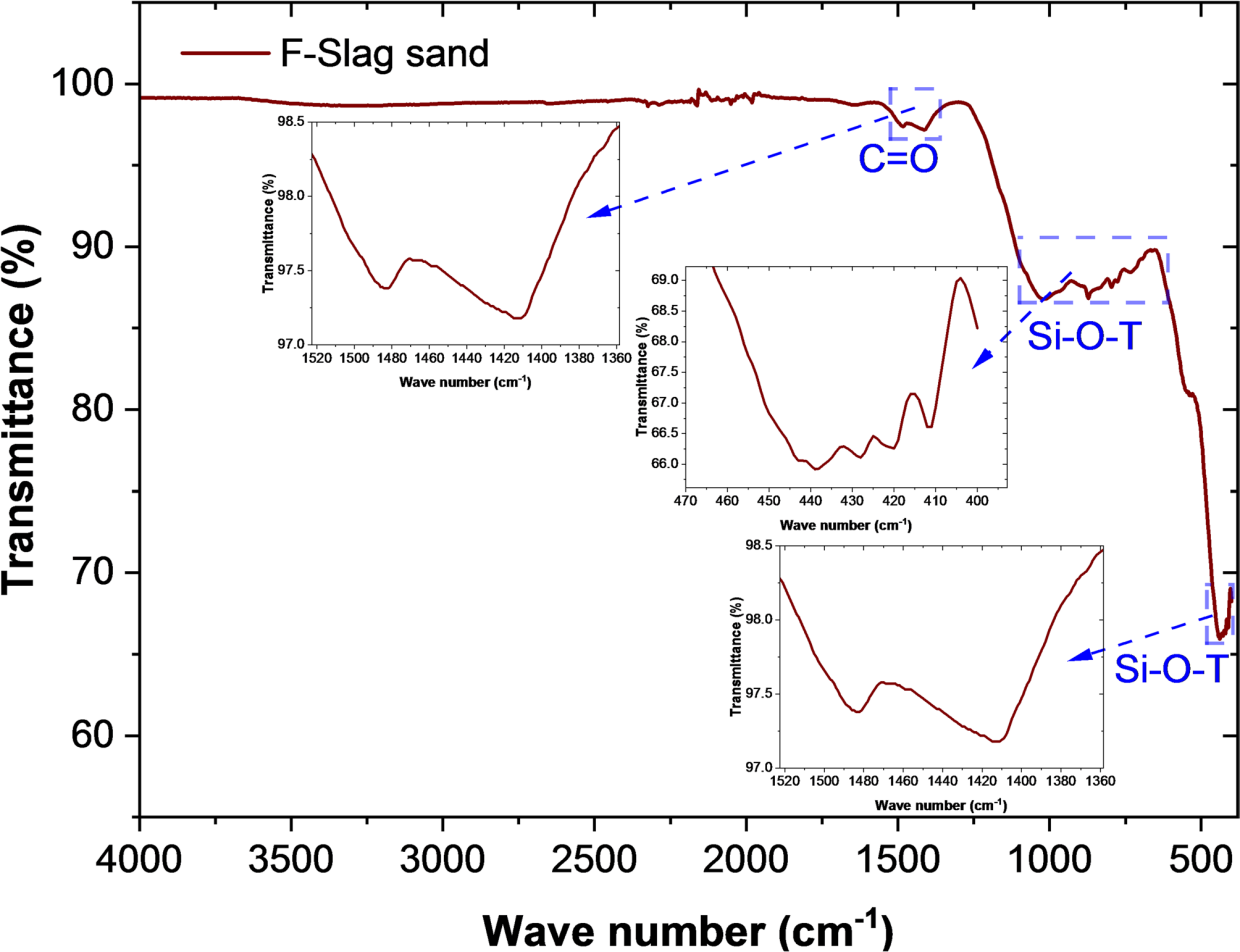
FTIR spectrum of F-Slag sand.

The FTIR spectrum of F-slag sand (Fig. 7) highlights the key functional groups associated with its mineralogical structure. A distinct absorption band around 1450 cm⁻¹ corresponds to the stretching vibrations of C = O groups, which can be attributed to the presence of CO_3_^2−^ showing the presence of carbonates^49^. The strong and broad bands observed in the region of 900–1100 cm⁻¹ are assigned to Si–O–T, where T represents tetrahedral Si or Al, asymmetric stretching vibrations, reflecting the presence of silicate and aluminosilicate phases, which are the dominant structural motifs in FA and GGBS. Ismail et al.^50^ reported a characteristic band at 978 cm^− 1^ in GGBS and 1090 cm^− 1^ in FA. Another sharp band near 450–500 cm⁻¹ is also attributed to Si–O–T bending modes, further confirming the silicate backbone of the material^51^. Collectively, these spectral features confirm that the F-slag sand primarily consists of silicate-rich phases with traces of carbonate phases. The prevalence of stable aluminosilicate structures supports the mineralogical insights obtained from XRD analysis (Fig. 5). It underscores the material’s suitability as an alternative fine aggregate, offering durability and chemical reactivity advantageous for construction applications.

**Fig. 8:**
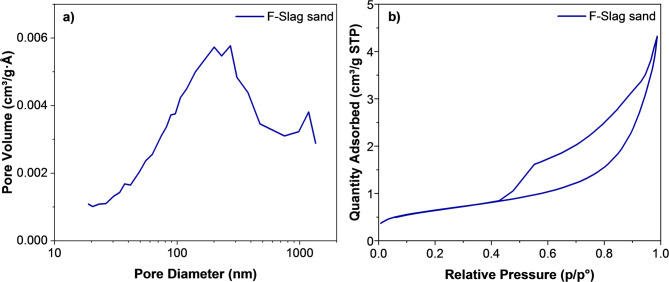
**a**) Pore size distribution curve **b**) Nitrogen adsorption-desorption isotherm of F-Slag sand.

The nitrogen pore size distribution with adsorption–desorption analysis of the F-slag sand is shown in Fig. 8a-b. The pore size distribution (Fig. 8a) shows a broad range of pores centered around 50–200 nm, extending into the macropore region. The total pore volume is in the range of 0.004–0.006 cm^3^/g·Å, while the BET surface area is measured as 2.264 m^2^/g. The higher water absorption (17%) of F-Slag sand particles, despite the relatively low BET surface area of 2.264 m^2^/g, can be attributed to the presence of interconnected meso- to macropores rather than a high proportion of micropores. The total pore volume of F-slag sand indicating that water uptake is primarily governed by pore volume and pore connectivity, which facilitate capillary water retention. In contrast, BET surface area mainly reflects the contribution of micropores, which play a limited role in bulk water absorption. Therefore, the observed high water absorption of F-slag sand is consistent with its pore structure characteristics rather than its specific surface area. The isotherm (Fig. 8b) exhibits a typical type IV profile with an H3 hysteresis loop^52,53^, indicating the predominance of mesopores with slit-like pore geometry formed by aggregated particles, which confirms a heterogeneous textural framework with coexisting mesopores and macropores. Such a relatively low surface area, combined with meso–macroporous connectivity, is favourable for construction applications, as it promotes mechanical interlocking within cementitious matrices rather than excessive water demand^54,55^. These features establish F-Slag sand as a promising alternative for fine aggregates with a balanced textural profile.

### Leaching analysis of F-slag sand

The concentration of PTEs measured in leachates derived from F-Slag sand is presented in Table 2, alongside the corresponding international regulatory limits for comparison. All measured concentrations of PTEs fall significantly below the permissible thresholds, revealing that F-Slag sand poses no environmental threat regarding heavy metal release. These findings reinforce the material sustainability for the applications like construction and mine void backfilling. The concentration (mg/l) of Ca, K, Mg, Fe, and Na cations is measured as 18.433, 24.408, 0.792, 1.817, and 58.332 respectively.

**Table 2:** Concentrations of potentially toxic elements in the leachates from F-Slag sand.

Constituent (mg/l)	F-Slag sand	Permissible limits (mg/l)				
		MoEF&CC ^34^	VROM ^35^	CONAMA ^36^	ABNT NBR ^37^	USEPA ^38^
As	0.042	5	2.1	10	1	5
Cu	0.001	25	140	60	-	100
Cr	0.025	5	5	5	5	5
Cd	0.002	1	1	1.3	0.5	1
Zn	0.084	250	1000	300	-	500
Pb	0.295	5	3.6	-	1	5
Ni	0.014	20	50	30	-	7
Hg	0.001	0.2	0.6	0.5	0.1	0.2

### Environmental risk assessment

In general, P_i_ ≥ 0.5 indicates potential ecological risk, and P_i_ > 1 indicates pollution. As such, higher P_i_ values reflect more severe contamination. Complementing this, the risk index (RI = ∑E_i_) categorizes ecological risk levels^39,40^. RI < 40 denotes low risk, 40–80 moderate, 80–160 considerable, 160–320 high, and RI > 320 indicates extreme ecological threat.

A detailed calculations of P_i_ and E_i_ are presented in Table 3. The P_i_ value of F-Slag sand is 0.04, which is well below the threshold (0.5) of concern, implying negligible impact on soil and water. Similarly, the overall RI value of 0.64 is substantially lower than even the lowest risk category, clearly affirming that F-Slag sand poses no ecological risk, and its potential environmental hazard can be safely disregarded.

**Table 3:** Environmental risk assessment results of F-Slag sand.

Heavy metal	Concentration (mg/kg) (C_i_)	Toxicity response factor (T_i_)	Standard reference value (S_i_) (mg/l)	Single factor index *P*_i_ = C_i_ / S_i_	Potential ecological risk index E_i_ = T_i_ × *P*_i_
As	0.042	10	15	2.80E-03	2.80E-02
Cu	0.001	5	35	2.86E-05	1.43E-04
Cr	0.025	2	90	2.78E-04	5.56E-04
Cd	0.002	30	0.2	1.00E-02	3.00E-01
Zn	0.084	1	100	8.40E-04	8.40E-04
Pb	0.295	5	35	8.43E-03	4.21E-02
Ni	0.014	5	40	3.50E-04	1.75E-03
Hg	0.001	40	0.15	6.67E-03	2.67E-01

### Potential applications of F-Slag sand

An effort is further made to propose novel applications of the developed F-Slag sand across construction and geotechnical domains. The performance assessment of F-Slag sand demonstrates its strong potential in construction applications. Gradation analysis confirms that T4 and T5 satisfy ASTM C33^41^ and IS 383^56^ (Zone 2) standards for concrete application while T2 and T3 comply Zone-1 sand category. Mortar requirements specified in IS 2116^57^ are precisely met by T4 and T5, and the fine fraction complies with the IS 1542^58^ specifications for plastering applications. In terms of macro-performance, the developed sand exhibits a crushing strength of 14.8%, well within the 30% limit prescribed for construction-grade aggregates^56^. Its relatively low density makes it advantageous for lightweight construction, reducing dead loads while maintaining structural efficiency^59^. Furthermore, thermal stability and meso–macroporous connectivity can enhance the durability and mechanical interlocking. Notably, the production of F-Slag sand valorises industrial by-products such as FA and GGBS, reinforcing circular economy principles by converting wastes into high-value added construction materials.

For mining applications, F-Slag sand performs exceptionally superior. Its gradation complies with particle size limits prescribed for mine void filling, hydraulic stowing, and pneumatic stowing, with fines limited to 10% below 0.075 mm and 15% in the 40–150 μm range^60^. The predominance of spherical particles well illustrates their flowability and placement efficiency^39,61^. These attributes make F-slag sand a manoeuvrable and efficient backfilling material, directly addressing the shortage of natural river sand in mine backfilling applications. By transforming industrial by-products into technically robust substitutes for natural aggregates, F-Slag sand not only meets engineering performance benchmarks but also contributes to sustainable resource management and circular economy practices.

Furthermore, the adoption of F-Slag sand aligns with global sustainability goals. By repurposing industrial by-products such as FA and GGBS, it supports SDG 12 (Responsible Consumption and Production) while its use as an alternative for river sand promotes SDG 9 (Industry, Innovation, and Infrastructure), highlighting its role in sustainable and eco-friendly construction practices.

## Conclusions

The present study demonstrates the development of artificial sand, replicating river sand, with the synergistic use of FA and GGBS. It is established that geopolymerization with pelletization techniques are profoundly successful in developing F-slag sand within the envisioned particle size range from 4.75 to 0.075 mm. Gradation analysis reveals that F-Slag sand encompasses 7–41% fine, 24–46% medium, and 12–56% coarse sand fractions. It is found that 100% binder solids are converted into sand sized fractions. It is importantly appreciated that the synergistic usage of GGBS and FA completely eliminated the necessity of elevated curing for F-slag sand. SEM analysis portrayed agglomeration of BS particles, forming F-Slag sand, occurred by binding and bonding mechanisms. 3D visualization of particles morphology (size, shape, and spatial distribution) revealed a uniformly dense internal structure with minimal voids of F-slag sand. The characterization of F-slag sand confirmed silicate-rich aluminosilicate structure, and meso–macroporous texture with BET surface area of 2.264 m²/g. Thermal analysis indicated a total mass loss of 4.07% when heated up to 800 °C. Leaching test results confirm that F-Slag sand is environmentally benign and non-hazardous. The environmental risk analysis affirms that F-Slag sand poses no ecological threat, reinforcing its potential as a sustainable alternative to natural river sand. These findings highlight the structural compactness and integrity of F-Slag sand, underscoring its potential for high-performance construction applications. The findings hold significant implications, as they lay an outline for optimizing future manufacturing processes by synergizing two distinct industrial by-products, FA and GGBS, for producing novel alternate fine aggregate, i.e. F-Slag sand.

Despite the promising results, the present study has certain limitations. The experimental investigations are conducted under controlled laboratory conditions using specific sources of FA and GGBS. Variations in chemical composition or fineness of these materials may influence sand formation dynamics and performance. Long-term durability aspects such as resistance to chemical attack, cyclic wetting–drying, and freeze–thaw conditions are not evaluated in this study. Additionally, large-scale production feasibility, cost economics, energy consumption during pelletization, and field performance of F-Slag sand in actual construction applications require further investigation. Future studies should address these aspects to fully establish the practical applicability and long-term reliability of F-Slag sand.

## Data Availability

The datasets generated and/or analysed during the current study are available from the corresponding author on reasonable request.
